# Effects of Daily Raspberry Consumption on Immune-Metabolic Health in Subjects at Risk of Metabolic Syndrome: A Randomized Controlled Trial

**DOI:** 10.3390/nu12123858

**Published:** 2020-12-17

**Authors:** Maximilien Franck, Juan de Toro-Martín, Véronique Garneau, Valérie Guay, Michèle Kearney, Geneviève Pilon, Denis Roy, Patrick Couture, Charles Couillard, André Marette, Marie-Claude Vohl

**Affiliations:** 1 Centre Nutrition, Santé et Société (NUTRISS) and Institut sur la Nutrition et les Aliments Fonctionnels (INAF), Université Laval, Québec, QC G1V 0A6, Canada; maximilien.franck.1@ulaval.ca (M.F.); juan.de-toro-martin.1@ulaval.ca (J.d.T.-M.); veronique.garneau@fsaa.ulaval.ca (V.G.); valerie.guay@fsaa.ulaval.ca (V.G.); michele.kearney@fsaa.ulaval.ca (M.K.); genevieve.pilon@criucpq.ulaval.ca (G.P.); denis.roy@fsaa.ulaval.ca (D.R.); patrick.couture@fmed.ulaval.ca (P.C.); charles.couillard@fsaa.ulaval.ca (C.C.); andre.marette@criucpq.ulaval.ca (A.M.); 2 School of Nutrition, Université Laval, Québec, QC G1V 0A6, Canada; 3 Quebec Heart and Lung Institute (IUCPQ) Research Center, 2725 Chemin Sainte-Foy, Québec, QC G1V 4G5, Canada; 4 Endocrinology and Nephrology Unit, CHU de Québec-Université Laval, Québec, QC G1V 4G2, Canada

**Keywords:** berry fruits, metabolic syndrome, multi-omics, immunity, gene expression, sphingolipids, phenolic compounds

## Abstract

Consumption of red raspberries has been reported to exert acute beneficial effects on postprandial glycemia, insulinemia, triglyceridemia, and cytokine levels in metabolically disturbed subjects. In a two-arm parallel-group, randomized, controlled trial, 59 subjects with overweight or abdominal obesity and with slight hyperinsulinemia or hypertriglyceridemia were randomized to consume 280 g/day of frozen raspberries or to maintain their usual diet for 8 weeks. Primary analyses measured metabolic differences between the groups. Secondary analyses performed with omics tools in the intervention group assessed blood gene expression and plasma metabolomic changes following the raspberry supplementation. The intervention did not significantly affect plasma insulin, glucose, inflammatory marker concentrations, nor blood pressure. Following the supplementation, 43 genes were differentially expressed, and several functional pathways were enriched, a major portion of which were involved in the regulation of cytotoxicity, immune cell trafficking, protein signal transduction, and interleukin production. In addition, 10 serum metabolites were found significantly altered, among which β-alanine, trimethylamine N-oxide, and bioactive lipids. Although the supplementation had no meaningful metabolic effects, these results highlight the impact of a diet rich in raspberry on the immune function and phospholipid metabolism, thus providing novel insights into potential immune-metabolic pathways influenced by regular raspberry consumption.

## 1. Introduction

Both clinical and epidemiological studies highlight the contribution of a plant-food-predominant diet in the maintenance, or even the improvement, of metabolic homeostasis owing to its fiber and phytochemical contents [1]. Long-term consumption of a diet poor in these components constitutes the main predisposing factor to metabolic homeostasis dysregulation [2,3]. Metabolic dysregulations can be seen as a broad range of intermediate phenotypes, all converging with time toward metabolic syndrome, a cluster of interrelated immune-metabolic abnormalities increasing the risk of developing type 2 diabetes or atherosclerotic diseases [4]. Although public health authorities promote plant-food-predominant diets [5], efforts to increase vegetable and fruit consumption have been hardly efficient. Accordingly, it has been demonstrated that dietary habits are difficult to change over the long term [6], highlighting the need to identify simple nutritional interventions that increase the fiber and phytochemical contents of the diet. In this regard, growing evidence has supported the role of berry fruits in the prevention and management of metabolic disorders [7]. Among the most commonly consumed berries, red raspberry (Rb; *Rubus idaeus* L.) is low in sugar and particularly rich in both fiber and phenolic compounds, mostly anthocyanins, and ellagitannins [8].

Collectively, in vitro and ex vivo research conducted with Rb extracts or purified components have revealed various antioxidative, anti-inflammatory, and metabolic properties through which Rb components may help treat or improve immune-metabolic abnormalities [9,10]. Several animal studies have confirmed these beneficial effects with both Rb components and the entire fruit [11,12,13]. Moreover, some of these studies brought to light the immunomodulatory effects of Rb phenolic compounds [14,15]. Altogether, these results are consistent with those observed with black raspberry (*Rubus occidentalis*), although of reduced amplitude for an equal amount, presumably attributable to its lower phenolic content [16,17]. Unlike the black raspberry, for which the health-promoting effects have been confirmed in numerous human studies [18,19], very few human randomized controlled intervention studies have been undertaken to assess the health impact of whole Rb or its components, and most of them looked only at the short-term metabolic impact [20,21,22,23,24,25].

In that respect, the aims of this randomized controlled clinical trial were to investigate the health effects of Rb consumption on immune-metabolic features in subjects at risk of developing metabolic syndrome and to delineate the mechanisms underlying these effects through transcriptomics and metabolomics. In the context of personalized nutrition, this holistic approach is part of the efforts undertaken to further understand the impact of foods or nutrients on metabolic syndrome.

## 2. Materials and Methods

### 2.1. Study Participants and Eligibility Criteria

**Figure 1:**
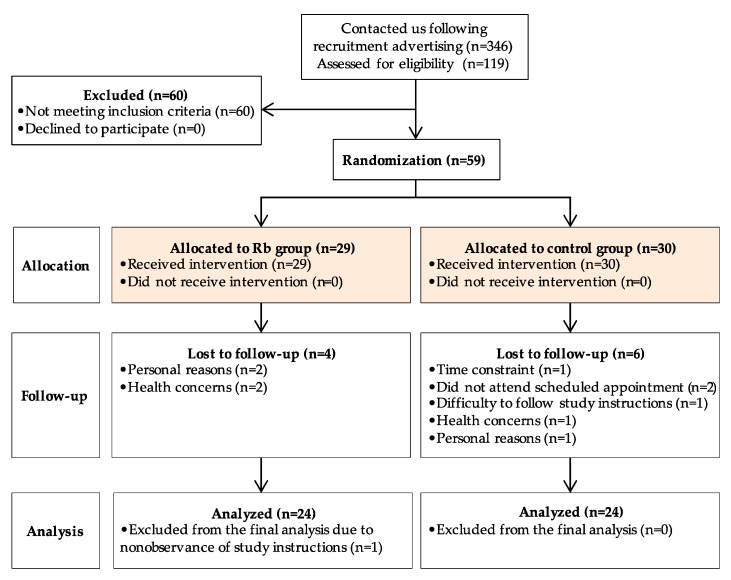
Patient flow diagram for recruitment, randomization, and data collection.

A two-arm parallel-group, randomized, controlled trial of the effects of Rb consumption on immune-metabolic parameters was conducted at the Institute of Nutrition and Functional Foods (INAF) at Université Laval between January 2018 and August 2019. Study participants from the greater Quebec City metropolitan area were recruited through emails, newspapers, and media advertisements. A total of 468 individuals contacted our research team for information related to the study, among which 119 were scheduled for a screening visit at the clinical investigation unit, and 59 were found to be eligible to participate in the study (Figure 1).

The age and medical history of participants were assessed at week 0, before the beginning of the intervention. To be included in the study, subjects had to be men or pre-menopausal women, Caucasian, aged between 18 to 60 years, have a body-mass index (BMI) between 25 and 40 kg/m^2^ or a waist circumference ≥ 94 cm for men and ≥80 cm for women, as well as meet at least one of the following criteria: Plasma triglycerides (TG) > 1.35 mmol/L, or insulin concentration > 42 pmol/L, using our new analytic method and corresponding to a threshold value of 60 pmol/L with the former method that was predictive of a higher risk of cardiovascular disease in the Quebec population [26]. Subjects were excluded if they had a diagnosis for diabetes, hypercholesterolemia, or hypertension, had a taste aversion for or were allergic or intolerant to Rb, were taking medication known to affect study parameters, had taken antibiotics, supplements, or natural health products on a regular basis over the past 3 months, had undergone surgery in the last 3 months, or had planned surgery during the duration of the study. Nicotine usage, non-conventional dietary patterns (e.g., vegan, gluten-free or ketogenic diets), bodyweight loss or gain greater than 5% in the last 3 months, or daily alcohol consumption greater than 2 drinks were also considered exclusion criteria. The study was approved by the Université Laval Ethics Committee in January 2018 (CER-Université 2017–218). Upon recruitment, written informed consent was obtained from all subjects prior to the beginning of the study procedures. This trial was registered at clinicaltrials.gov as NCT03620617. Changes in anthropometric and metabolic variables in response to the Rb supplementation were defined as the primary outcomes, whereas pre- and post-supplementation changes in metabolomics and transcriptomics were considered as the secondary outcomes.

### 2.2. Nutritional Intervention

**Figure 2:**
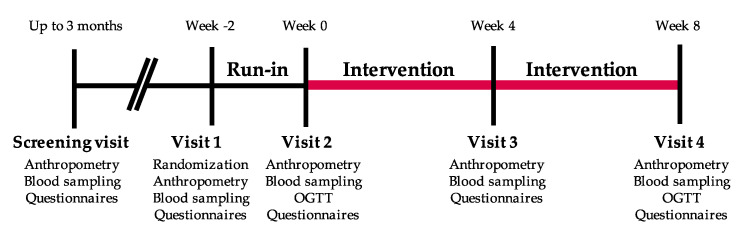
Graphical representation of the study protocol.

The randomization occurred after eligibility was confirmed. Participants were randomized to study arms using an in-house electronic management platform. A sex-stratified block randomization ensuring a 2:2 treatment allocation for each sex to either the intervention group consuming frozen Rb, or the control group was used (Figure 2). The randomization process was under the responsibility of the lead study coordinator. Three clinical coordinators and one graduate student were responsible for the recruitment and follow-up of participants in this study. None of the participants dropped out when they found out which group they had been assigned to. After a 2-week run-in period, participants assigned to the intervention group were invited to consume 280 g of frozen Rb daily (roughly 2 cups) for 8 weeks. Control group participants were invited to maintain their health and food habits stable for an 8-week period. Participants of both groups were scheduled for clinical visits after the run-in period (week 0), during the intervention (week 4), and at the end of the protocol (week 8). During the protocol, including the run-in period, participants were asked to avoid the use of supplements, natural health products, wine, or products with a polyphenolic profile similar to that of Rb and to limit the consumption of berries other than those provided, and any other products containing berries, to 2 portions per week. They were also instructed to limit coffee and tea consumption to 1 serving/day, and alcohol to 2 drinks/week. Participants had to complete a journal reporting any deviation from the dietary instructions listed above, as well as the Rb quantity consumed for the intervention group. The count of empty Rb packages returned by the participants was used to assess their compliance with the study protocol. Participants received regular emails to follow-up on any issues that may arise and to enhance adherence to the protocol.

### 2.3. Anthropometric Measurements

At each clinical visit, waist and hip circumferences were measured to the nearest millimeter according to procedures recommended at the Airlie conference [27]. Height was also measured to the nearest millimeter. Bodyweight was measured using a BWB-800 electronic scale (Tanita, Arlington Heights, IL, USA) to an accuracy of 0.1 kg. For weight measurements, participants were asked to wear light indoor clothes and to remove their shoes. BMI was calculated as weight in kilograms divided by height in meters squared (kg/m^2^). Systolic (SBP) and diastolic (DBP) blood pressure measurements were assessed while sitting on a chair after a 10 min rest. The mean of 3 measurements performed at 3 min intervals was used for the analyses.

### 2.4. Nutritional and Physical Activity Assessments

Nutritional habits were assessed at each clinical visit to the clinical investigation unit through the use of a web-based and self-administered Food Frequency Questionnaire (FFQ), validated for French-speaking Canadian adults [28]. The FFQ was based on 136 items grouped into the 8 following categories: Dairy products, fruits, vegetables, meat and alternatives, cereals and grain products, beverages, other foods, and supplements. Participants reported how often they consumed each item per day, per week, per month, or none at all in the last month. Finally, pictures with examples of portion sizes were used to reflect a better estimation of the portion consumed by the participant. Physical activity habits were evaluated using the Leisure Time Activity questionnaire, which was completed by participants at the beginning and at the end of the intervention [29].

### 2.5. Biochemical Analyses

Fasting plasma samples were collected on weeks 0, 4, and 8, and used to measure total-cholesterol (Total-C), LDL-cholesterol (LDL-C), HDL-cholesterol (HDL-C), TG, glucose, and insulin levels. Total-C and TG concentrations were measured using enzymatic assays [30]. Apolipoprotein B (ApoB) was measured by immunonephelometry assay using a Siemens Dimension Vista™ 1500 Analyzer. The HDL-C fraction was obtained after precipitation of VLDL and LDL particles in the infranatant with heparin manganese chloride. LDL-C was calculated with the Friedewald formula [31]. High-sensitivity C-reactive protein (CRP) levels were measured by immunoassay [32]. Fasting glycated hemoglobin (HbA1c) was measured in plasma by immunoturbidimetry (Biorad HPLC D-100). Oral glucose tolerance tests (OGTTs) using a 75 g glucose solution were conducted twice during the intervention period (weeks 0 and 8). Blood samples were drawn at −15, 0, 15, 30, 60, 90, and 120 min during the OGTT to assess plasma insulin and glucose levels. Glucose concentrations were enzymatically measured [33], while insulin concentrations were measured by chemiluminescence (Siemens Advia Centaur XPT). The homeostatic model assessment of insulin resistance (HOMA-IR) index and the Matsuda index were calculated from OGTT values [34,35].

### 2.6. Transcriptomic Analyses

Gene expression profiling before and after the supplementation was conducted in whole blood, commonly used to assess the effects of dietary interventions in human trials [36]. The effects of Rb supplementation on the gene expression profile was examined in blood samples collected into PAXgene preparation tubes (Qiagen, Valencia, CA, USA) at week 0 and 8, and stored at −80 °C until the analyses. Total RNA was extracted, and samples were sent to the Génome Québec Innovation Centre (McGill University, Montreal, Canada) for sequencing. The purity and integrity of RNA samples were assessed using a 2100 Bioanalyzer (Agilent, Santa Clara, CA, USA). RNA samples were converted to cDNA with the Illumina NEB stranded mRNA library preparation kit (Illumina, San Diego, CA, USA) based on the manufacturer’s protocol. The prepared libraries were then sequenced on an Illumina NovaSeq6000 S4 sequencer using paired-end, 100 bp reads. Quality trimming of the raw reads was carried out to remove bases with a Phred33 score < 30 and reads with less than 50 bases using Trim Galore (v0.6.5), a wrapper tool around Cutadapt (v1.15) and FastQC (v0.11.9). Kallisto (0.46.2) with 100 bootstraps and default parameters was run for alignment and normalization. Reads were aligned to the GRCh38 human reference transcriptome [37]. Transcriptomic data reported as counts per million (cpm), were filtered based on a worthwhile number of counts in a minimum number of samples in the edgeR v3.28.1 package [38]. The differential expression analysis was conducted using a generalization of a paired t-test implemented in the quasi-likelihood functionality of edgeR. Differentially expressed transcripts between pre- vs. post-intervention at a nominal *p*-value < 0.05 were considered for further analysis. The functional significance of genes showing at least a 25% difference (1.25-fold change) between pre- and post-supplementation states was explored by pathway enrichment analysis using the clusterProfiler v3.16.0 R package [39]. The clusterProfiler package implements statistical methods to analyze and visualize functional profiles of genes and gene clusters and produces adjusted *p*-values using the Benjamini–Hochberg procedure (BH-p) to indicate pathways that were significantly enriched. The following pathway databases were used for functional enrichment analysis: Gene Ontology Biological Processes (GO-BP) and the Kyoto Encyclopedia of Genes and Genomes (KEGG). Transcriptomic data analysis was implemented in R v3.6.3.

### 2.7. Metabolomic Analyses

The quantitative analysis of 630 metabolites from 26 biochemical classes was performed in paired plasma samples from 24 participants before and after the Rb supplementation with the MxP^®^ Quant 500 kit (Biocrates Life Sciences AG, Innsbruck, Austria). The targeted metabolomic profiling was performed by the Analytical Facility for Bioactive Molecules at the Hospital for Sick Children (Toronto, Canada). Metabolite data from 24 matched participants were processed using the MetaboAnalystR package (v3.0) [39]. First, 117 features with a constant or single value across samples were found and deleted. Non-informative signals were further filtered out based on the interquartile range estimate, and samples were normalized by quantile normalization. Metabolite data were log-transformed and scaled by Pareto scaling. After quantile normalization, one more feature with a constant value was found and deleted. From the original 630 metabolites analyzed, a total of 382 were finally retained for statistical analysis. Paired t-tests were used to analyze within-subject changes in plasma metabolite levels between pre- and post-supplementation states. A volcano plot associated with paired t-tests was further used to identify the most differentially abundant metabolites between pre- and post-supplementation groups. A *p*-value < 0.05, along with a count of significant pairs higher than 50% showing at least 25% difference (1.25-fold change), were the criteria used to consider metabolite plasma levels differing significantly between pre- and post-supplementation states. The dimensional reduction was conducted by using the partial least squares discriminant analysis (PLS-DA), a supervised algorithm able to reduce the number of metabolites in high-dimensional metabolomics data to produce robust and easy-to-interpret models. This method was able to differentiate the class membership through multivariate regression of a given set of metabolites. In the present study, we used a variation of PLS-DA, the multilevel PLS-DA (mPLS-DA), given its ability to exploit the paired structure of the multivariate data obtained before and after the Rb supplementation in the same group of participants [39,40]. Concretely, a sparse mPLS-DA (smPLS-DA) was used to identify the most important metabolites helping to discriminate matched study groups [41]. The smPLS-DA algorithm was implemented using the mixOmics R package (v6.12.1) [42]. Variable importance in projection (VIP) coefficients were computed as a weighted sum of squares of the smPLS-DA loadings to depict the relative importance of each metabolite in the classification model. Predictors with large VIP were the most relevant for discriminating class membership [43].

### 2.8. Statistical Analyses

The normality of distribution of all variables was assessed using skewness and kurtosis, and none of the variables needed transformation before analysis. Descriptive characteristics between the Rb and the control group were presented as means (±SD) or frequencies. Chi-square tests for categorical variables and analyses of variance (general linear model, type III sum of squares) for continuous variables were used to seek for inter-group differences in baseline characteristics and in changes between follow-up and baseline. The mixed procedure in SAS with special provisions for repeated measures that incorporated data from all visits was used to examine the effects of time, treatment, and their interaction on clinical variables. Data were adjusted for age, sex, BMI, and baseline values. For all analyses, a *p*-value < 0.05 was considered for statistical significance. Statistical analyses were performed using SAS version 3.8 (SAS Institute, Cary, NC, USA).

## 3. Results

### 3.1. Trial Flow, Baseline Characteristics, and Compliance

Of the 119 subjects screened for eligibility, 59 were enrolled in the study, 29 were randomized to the Rb group, and 30 to the control group (Figure 2). Eleven participants (6 and 5 from the Rb and control group, respectively) withdrew during follow-up by the inability to comply with study procedures or schedule conflict. Therefore, 48 subjects were included in the per-protocol analysis.

According to BMI values, 22 subjects had obesity (BMI > 30 kg/m^2^), 19 were overweight (BMI between 25 and 30 kg/m^2^), and 7 were in the normal weight range (BMI < 25 kg/m^2^). The mean BMI of participants was 29.9 kg/m^2^ (ranging from 22.3–43.7), and the mean age was 32.2 years (ranging from 22 to 57). Upon randomization, baseline characteristics were well-distributed at baseline and, therefore, no significant differences between the Rb group and the control group were observed for age, sex, anthropometric, physiologic, or metabolic variables (Table 1). However, although not statistically significant, the HOMA-IR difference between groups may be considered as clinically meaningful. No adverse events related to Rb supplementation were reported during the study. Adherence to the study protocol was good, with an overall compliance of 92.8%.

### 3.2. Food Intake and Physical Activity

There was no significant difference in energy intake between groups at any time point during the protocol. Accordingly, no significant treatment by time interactions was found in macronutrients or other nutritional compounds, with the exception of those deriving directly from Rb composition, namely glucose, fructose, soluble and insoluble fiber (Table 2). According to the FFQ data, adding 2 cups/day of Rb accounted, respectively, for 17.6%, 17.1%, 13.3%, and 25.5% of total dietary glucose, fructose, soluble, and insoluble fibers intakes. Moreover, when analyzing dietary intakes based on food groups (bread and cereals, fruits, vegetables, dairy products, animal proteins), there were no significant differences between groups except for fruit consumption (*p* < 0.0001, a mean of 2, 3 portions for the control group vs. 6 portions for the Rb group during the intervention). The intervention resulted in a nutritionally significant shift (around 3 servings) in fruit consumption in the Rb group. Physical activity remained stable throughout the protocol (Table 2).

### 3.3. Primary Outcomes

We found no significant differences between follow-up and baseline values between control and intervention groups for any of the parameters analyzed (Table 3). The statistical adjustment for age, sex, and BMI had no effect on the lack of differences between the Rb and control groups (data not shown).

Repeated measures analysis revealed a significant treatment by time interaction effect on SBP (*p* = 0.03) and plasma ApoB levels (*p* = 0.03) (Table 4). Indeed, in the control group, we found a significant increase in plasma ApoB concentrations from the beginning of the intervention (0.86–0.16 g/L) to the mid-point visit (0.95–0.17 g/L), before regaining its initial level at the end of the study (0.87–0.19 g/L). Again, a significant effect of time was observed for both HDL-C (*p* = 0.03) and LDL-C (*p* = 0.01) in the control group. There was no other singular or interaction effect of time and treatment for any other metabolic or anthropometric variable. In accordance to the lack of change in fasting and postprandial plasma insulin and glucose concentrations in response to the intervention, both HOMA-IR and MATSUDA indexes derived from OGGT were not found to differ significantly.

### 3.4. Secondary Outcomes

#### 3.4.1. Effects of Rb Supplementation on Gene Expression

**Figure 3:**
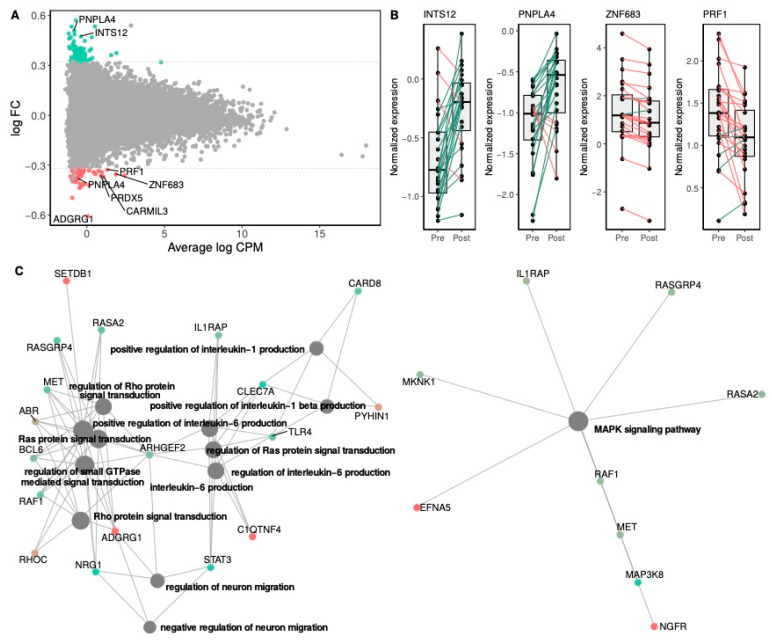
Gene expression change between pre- and post-supplementation states in the Rb group. Panel (**A**) shows the log2 average abundance of transcripts in counts per million mapped reads (log CPM) on the x-axis and the log2 fold-change (log FC) on the y-axis. Non-significant genes are represented by grey dots. Over- and under-expressed genes (FC > 1.25) with unadjusted significant differences (paired t-test *p*-value < 0.05) are colored in green and red, respectively. Significant differentially expressed genes from paired t-tests (*p*-value < 0.001) and showing at least a 1.25 FC are labeled with gene names. The dashed lines represent 1.25 FC. Panel (**B**) shows the top differentially expressed genes between pre- and post-supplementation states in the Rb group. Box and whisker plots show median, first, and third quartiles, and maximum and minimum values for the 24 sample pairs before (Pre) and after (Post) the Rb supplementation. The three transcripts, which exhibited the most significant (*p*-value < 0.001) over- and under-expression derived from paired *t*-tests (post vs. pre), are shown on the top and bottom rows, respectively. Green and red lines stand for increasing or decreasing gene expression levels between pre- and post-supplementation states within individual paired samples. Panel (**C**) shows network plots of enriched terms following the Rb supplementation. Network plots depict the linkages among differentially regulated gene clusters and functional enriched terms in the Gene Ontology Biological Processes (GO-BP) (left) and Kyoto Encyclopedia of Genes and Genomes (KEGG) (right) pathway databases. The size of the grey dots is proportional to the number of genes in the enriched pathway (from 7 to 13 genes), and the red-to-green color gradient of gene dots represents the direction of the gene expression fold-change following the Rb supplementation.

The study of differential gene expression in blood cells in the Rb group revealed a total of 1384 genes differentially expressed following Rb supplementation (*p* < 0.05), from which 119 showed a fold change greater than 1.25. Following multiple testing correction, none of these genes reached the significance threshold of FDR-*p* < 0.05. We then relaxed the significance criteria in order to identify those genes differentially expressed at *p* < 0.001 (Table 5). A total of 43 genes were identified, among which 9 were found to be upregulated and 34 downregulated, with fold changes ranging from −1.5 (*ADGRG1*) to 1.4 (*PNPLA4*), with 8 of the 43 genes demonstrating at least a 1.25-fold change (Figure 3A). Individual changes in gene expression levels for the top differentially up- and down-regulated genes presented in Figure 3B illustrate the inter-individual variability of gene expression changes among participants.

The pathway enrichment analysis was conducted with all of the 119 genes showing at least a *p*-value < 0.05 and a fold change > 1.25 in response to the Rb supplementation (Figure 3C). Most differentially expressed genes were clustered into intracellular signal transduction pathways, as shown by the top-12 significantly enriched GO-BP categories, including those involved in the regulation of Ras and Rho signal transduction and several pathways related to the production of interleukins IL-6 and IL-1β. A relevant signal transduction pathway related to MAPK signaling was also found to be significantly enriched in the KEGG database.

#### 3.4.2. Effects of Rb Supplementation on Metabolite Levels

**Figure 4:**
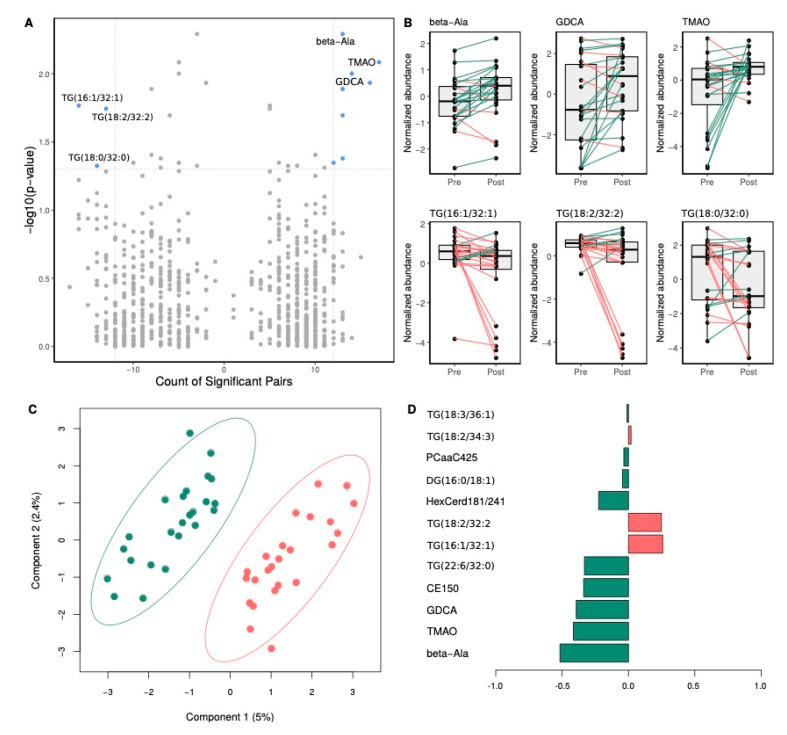
Impact of Rb supplementation on plasma metabolite levels. Panel (**A**) shows a volcano plot of paired comparisons between metabolite plasma levels in pre- and post-supplementation groups. On the x-axis, a count of significant sample pairs is shown. On the y-axis, the minus logarithm of paired t-test *p*-values is shown. Metabolites showing statistically significant changes following the Rb supplementation (*p*-value < 0.05 and fold change > 1.25) are depicted as blue dots on the right (increase) and left (decrease) top corners. Top-three significantly different metabolites of both increasing and decreasing values are labeled. Panel (**B**) shows top metabolites showing significant changes following Rb supplementation. Box and whisker plots show median, first, and third quartiles, and maximum and minimum values for the 24 sample pairs before (Pre) and after (Post) the Rb supplementation. The three metabolites, which exhibited the most significant decrease and increase following the supplementation, are shown on the top and bottom rows, respectively. Green and red lines stand for increasing or decreasing plasma metabolite levels between pre- and post-supplementation states within individual paired samples. Panel (**C**) shows a bi-dimensional score plot depicting the distinct plasma metabolomic profile between pre- (red dots) and post-supplementation (green dots) paired participants. The two principal components of the smPLS-DA model along with their corresponding variance in group discrimination are shown on y- and x-axis, respectively. Panel (**D**) shows a loading plot representing the top 10 metabolites selected on the first component of the smPLS-DA model. Most important metabolites in group discrimination are ordered according to their loading weights (horizontal bars), from bottom to top. Bar color indicates the group for which the mean value is the highest for each feature (orange and green stand for pre- and post-supplementation groups, respectively).

Paired *t*-test analysis brought out that, following Rb supplementation, a total of 14 metabolites had significantly different plasma levels (paired t-test *p*-value < 0.05), as compared to pre-supplementation levels. This number was reduced to 10 metabolites after applying more restricted statistical significance criteria (fold-change >1.25 and significant metabolite counts > 50%) (Figure 4A and Table 6). Plasma metabolites showing a significant reduction following Rb supplementation are shown on the top-left corner of the paired volcano plot, and those showing a significant increase are shown on the top-right corner (Figure 4A). Top-three under- and over-abundant metabolites are shown in paired boxplots in Figure 4B. More specifically, we identified β-alanine, deoxycholic acid glycine conjugate (GDCA), and trimethylamine N-oxide (TMAO) among the metabolites showing the most significant increase following Rb supplementation. On the other hand, three TG species, TG (16:1/32:1), TG (18:2/32:2) and TG (18:0/32:0), were among the metabolites undergoing a significant decrease (respectively –0.96 ± 1.83, –1.04 ± 1.99 and –0.93 ± 2.17 μmol/L, *p* = 0.02, 0.02 and 0.04). The score plot derived from smPLS-DA shows the complete separation of pre- and post-supplementation groups, without overlap (Figure 4C). Component 1 was the main one responsible for group discrimination, accounting for 5% of the variance, with component 2 accounting for 2.4%, respectively. The 10 metabolites associated with the first component and underlying the discrimination between pre- and post-supplementation groups are shown in the loadings panel of the first component (Figure 4D). Most of these metabolites were also identified as differentially abundant between pre- and post-supplementation groups (Figure 4B).

## 4. Discussion

To our knowledge, this is the first randomized controlled trial to investigate immune-metabolic changes in response to Rb supplementation over such a long timescale. Briefly, the present intervention resulted in the modulation of the expression of numerous genes and circulating levels of some plasma metabolites being potential biomarkers of metabolic syndrome. Despite the effect of Rb supplementation at transcriptomic and metabolomic levels, its impact on traditional metabolic syndrome features was relatively modest, with no significant effects observed on relevant metabolic outcomes. However, very few clinical trials have looked at the health effects of Rb, and most of them were based on acute postprandial interventions with small numbers of subjects. That way, findings from two recent studies indicated that acute Rb supplementation, with daily consumptions of 125 and 250 g, may lower postprandial hyperglycemia, hypertriglyceridemia, and inflammatory response (IL-6 and TNF-α), as well as SBP when extended for four weeks, in diabetic or prediabetic individuals [20,21]. By contrast, two other acute postprandial studies conducted on healthy subjects offered less conclusive results, with no lowering effects on glycemic and insulinemic responses [23,24]. Overall, results from clinical trials suggest that Rb consumption can acutely attenuate metabolic syndrome features in subjects with a pre-existing metabolic condition, lowering postprandial glucose, plasma TG and inflammatory biomarkers levels. The mild clinical impact of the present Rb supplementation is, therefore, not surprising and may be attributable to the fact that participants were at risk of developing metabolic syndrome but displayed limited metabolic alterations. Thus, it appears that Rb effects may be clinically noticeable on individuals whose homeostatic set points have shifted to altered states. However, the significant increase in glucose and fructose intake observed in the Rb group, and attributable to the reported increase in fruit servings, might have masked the effects of Rb on cardiometabolic health.

We further used transcriptomics and metabolomics to reveal the effects of Rb at a molecular level. Two of the most significantly down-regulated genes, *ZNF683* and *ADGRG1*, are both linked to immune system functioning. *ZNF683* encodes for a key regulatory transcription factor of natural killer (NK) cell differentiation [44] that also drives the expression of *ADGRG1* [45]. Furthermore, *ADGRG1* is down-regulated upon NK cell activation, controls NK cell effector functions, and is thus closely associated with the production of cytolytic proteins, such as perforin and granzyme, which are major players of the cytolytic process of NK cells and T lymphocytes [45]. Accordingly, *PRF1*, *GZMH*, *GNLY*, *FGFBP2,* and two transcript variants of *NKG7*, which code, respectively, for the perforin-1 protein, granzyme H, granulysin, fibroblast growth factor-binding protein 2, and NK cell granule protein 7 were down-regulated. In addition, the gene encoding for the sphingosine-1-phosphate receptor 5 (*S1PR5*), which is implicated in monocyte trafficking and the exit of mature NK cells from the bone marrow and lymph nodes into blood and lymph circulation, was also significantly downregulated [46,47]. S1PR5 belongs to the G protein-coupled receptor family S1PR1-5, known to mediate the effects of sphingosine-1-phosphate (S1P), a bioactive phospholipid, which exerts pleiotropic effects. S1P stems from the phosphorylation by sphingosine kinases of sphingosine that is derived from the hydrolysis of ceramide species [48]. With the class of hexosylceramides (HexCer) being involved in the extensive network of sphingolipids metabolism [49], it is worth highlighting the potential connection between S1P and HexCer(d18:1/24:1), a ceramide whose plasma levels significantly increased in response to the intervention. Similarly, GDCA, another metabolite undergoing a significant increase following the intervention, has been shown, amongst other conjugated bile acids, to activate S1PR2, which belongs to the receptor family mentioned above [50].

Several signaling pathways related to IL-1β and IL-6 production, as well as to Ras, Rho, and MAPK protein signal transduction, were found to be significantly enriched in response to Rb supplementation. Interestingly, Ras, Rho, and MAPK are part of the signal transduction pathways on which S1PR1-5 action relies [51], suggesting a link with the downregulation of *S1PR5*, as well as with the increase of plasma HexCer(d18:1/24:1) and GDCA. Taken together, this points toward a slight modulation of the “inside-out signaling” process of immune cells. Of interest, animal studies have demonstrated beneficial effects of black raspberry on colorectal and pancreatic cancer, in conjunction with enhanced infiltration of NK cells into the colon and modulations of cytotoxic NK and T cell subsets [16,52]. Part of Rb effects has been linked to its richness in ellagitannins and anthocyanins. Previous research has shown that ellagitannins can modulate mice and human leucocytes activity and cytokine production [14,53]. Based on these findings, part of the transcriptional variations observed herein could be attributed to the high ellagitannins content of Rb.

Amongst metabolites tested, β-alanine exhibited the largest increase following Rb supplementation. It is the intermediate between glycine and gamma-aminobutyric acid (GABA), and acts as a partial GABA receptor antagonist. GABAergic signaling is known to play a role in gastrointestinal motility through vagus nerve stimulation [54]. Components of the GABA signaling machinery are also expressed in immune cells and are involved in the regulation of cytokine production, immune cell proliferation, and chemotaxis [55]. Moreover, we found that *GABBR1*, which is coding for the subunit 1 of GABA type B receptor, was the most upregulated gene in response to Rb. Interestingly, an agonist of the GABAB receptor has been shown to reduce the secretion of cytokines, including TNF-α and IL-6, in blood mononuclear cells and to interfere with their chemotaxis [56]. Based on these findings, one may speculate that the significant increase in plasma β-alanine concentration is causally related to *GABBR1* upregulation, as well as to IL-6 pathways enrichment. Surprisingly, TMAO, a gut microbiota-derived metabolite from dietary choline, choline-containing phospholipids, betaine, and carnitine, also underwent a large increase. It has been previously found that TMAO increases monocyte recruitment and activation, leading to the upregulation of inflammatory cytokine genes, monocyte adhesion, and to the formation of foam cells [57]. TMAO is then considered as a pro-atherogenic metabolite associated with an increased risk of cardiovascular disease. This increase in TMAO may be owed to an increase in animal protein consumption of the participants, which was not the case in the present study. More startling is that the reverse trend is generally observed with small fruit extract supplementation, which suggests the effect of an unknown factor that remains to be identified [58,59]. Finally, in addition to GDCA and HexCer (d18:1/24:1), other lipids belonging to cholesterol esters and glycerophospholipids pathways significantly increased, whilst triacylglycerols increased for some and decreased for others. Similarly, in a randomized controlled trial conducted on individuals with metabolic syndrome who consumed 300 g/day of berries comprising 100 g of Rb for 8 weeks, serum lipidomic profiling revealed several lipids discriminating berry consumers from controls, notably cholesterol esters, phosphatidylethanolamines, phosphatidylcholines, and TGs [24]. Of note here, are the shared biochemical pathways undergoing modulations following both interventions, first the cholesterol ester and triacylglycerols pathways, but also the functional connections between the phosphatidylcholines, TMAO, sphingosine and hexosylceramides. Our findings, together with those of Puupponen-Pimiä et al. [24], point toward sphingolipids and choline metabolism. Accordingly, studies linked sphingolipid metabolism dysregulation to insulin resistance and ceramide accumulation to insulin signaling inhibition [60]. In addition, an interconnection between polyphenols intake and sphingolipid metabolism has been established [61]. Specifically, anthocyanins were reported to attenuate insulin resistance by modulating sphingomyelin conversion and ceramide de novo synthesis [62], while ellagic acid has been identified as a potential inhibitor of sphingosine kinase [63].

Taken together, clinical, transcriptional, and metabolomical prisms have shed some light on the potential mechanisms underlying the health effects of Rb. A non-negligible part of these transcriptional results points toward slight modulations in the tight interplay between the cytotoxicity of lymphocytes and immune cell trafficking. This phenomenon may be viewed in relation to changes in phospholipid metabolism, *S1PR5* downregulation being potentially linked to the modulation of sphingolipid and choline metabolism, reflected in the upregulation of HexCer (d18:1/24:1) and TMAO, respectively. In addition, according to the heterogeneity observed in the response of genes and metabolites, these results point to the need for further research on the determinants of the inter-individual variability in response to foods and nutrients.

### Strengths and Limitations

This study has several limitations. First, participants were Caucasians at risk of metabolic syndrome, which limits the generalization of the results. Second, the use of a crossover instead of a parallel design could have reduced the inter-individual variability in baseline characteristics. In addition, freezing small fruits could alter their content in phenolic compounds and, therefore, reduce their effects. For instance, it has been reported that the anthocyanin content of Haskap berries was reduced by nearly 60% after being frozen at −18 °C for 6 months [64]. Due to high hemolysis in some plasma samples, which prevents adequate measurement of plasma insulin levels, HOMA-IR and Matsuda indexes have been calculated for only a limited number of subjects in each group. Finally, the free-living design of the present study led to a significant increase in fruits consumption in the Rb group. Accordingly, glucose and fructose intakes were significantly higher in the Rb group, as compared to the control group, thus limiting the possibilities of clearly delineating the effects of raspberries on cardiometabolic health. Nonetheless, this study has strengths such as its randomization, which prevents selection bias in allocating intervention to participants. Compliance to the treatment was high, with no differences observed between groups in withdrawals from the study. Thus, although a per protocol analysis was used, exclusion of data are unlikely to have introduced bias.

## 5. Conclusions

In conclusion, clinical results are in line with those previously reported in human studies [24,25], as it appears that Rb health effects are clinically noticeable only in subjects with advanced metabolic disorders [20,21]. Omics profiling provided an overview of Rb health effects at the molecular level. Future investigations should be deepened to increase our understanding of the impact of Rb consumption on phospholipid metabolism and immune system and the links in-between.

## Figures and Tables

**Table 1:** Clinical and laboratory baseline characteristics of study participants.

Variable	N	Control			N	Rb			*p*-value
Sex (men/women)	24	9/15			24	7/17			0.54
Age (years)	24	31.92	±	8.05	24	32.46	±	10.12	0.83
BMI (kg/m^2^)	24	29.38	±	3.94	24	30.42	±	5.00	0.43
Waist circumference (cm)	24	98.10	±	11.81	24	98.53	±	13.32	0.90
Fasting glucose (mmol/L)	24	4.84	±	0.47	23	4.98	±	0.59	0.38
Fasting insulin (pmol/L)	22	73.91	±	33.57	19	83.11	±	43.54	0.21
HbA1C (%)	24	5.05	±	0.29	24	5.03	±	0.31	0.85
Plasma TG (mmol/L)	24	1.56	±	0.78	24	1.46	±	0.80	0.66
ApoB-100 (g/L)	24	0.88	±	0.17	24	0.92	±	0.24	0.48
HDL-C (mmol/L)	24	1.33	±	0.23	24	1.32	±	0.40	0.94
LDL-C (mmol/L)	24	2.55	±	0.74	24	2.65	±	0.86	0.69
CRP (mg/L)	24	2.72	±	2.82	23	2.68	±	2.34	0.95
HOMA-IR	13	1.78	±	0.76	11	2.84	±	1.73	0.06
MATSUDA	13	5.79	±	2.43	11	4.54	±	2.24	0.21

Data are means ± standard deviations. Sex is presented as counts. *p*-values were obtained by a general linear model, type III sum of squares, and chi-square tests (sex). BMI, body mass index. HbA1C, glycated hemoglobin. TG, triglycerides. ApoB-100, Apolipoprotein B. HDL-C, high-density lipoprotein cholesterol. LDL-C, low-density lipoprotein cholesterol. CRP, C-reactive protein. HOMA-IR, homeostatic model assessment of insulin resistance.

**Table 2:** Daily dietary intakes by nutrients and by food group over time within and between groups.

Time	Week 0						Week 4						Week 8						*p*-Values		
Treatment	Control			Rb			Control			Rb			Control			Rb			Time	tx	tx*Time
Energy (kcal)	2132.8	±	823.2	2059.7	±	662.1	1992.7	±	692.4	1983.3	±	682.1	2060.7	±	638.7	2075.8	±	623.2	0.32	0.70	0.83
Lipids (g)	93.5	±	44.9	82.7	±	28.6	87.7	±	40.9	76.4	±	31.8	91.4	±	37.7	80.9	±	28.7	0.25	0.60	0.99
Proteins (g)	90.1	±	37.3	87.2	±	30.9	87.4	±	32.1	84.0	±	30.1	91.9	±	28.6	88.3	±	30.8	0.53	0.68	1.00
Soluble Fiber (g)	8.3	±	4.1	7.7 ^a^	±	3.3	7.3 ^x^	±	3.4	8.5 ^y^	±	3.1	7.5 ^x^	±	3.1	8.9 ^by^	±	3.1	0.69	0.01	0.02
Insoluble Fiber (g)	15.6	±	7.8	15.0 ^x^	±	6.8	13.8 ^a^	±	6.9	23.1 ^by^	±	6.6	14.4 ^x^	±	6.2	23.8 ^by^	±	6.3	<0.0001	<0.0001	<0.0001
Fructose (g)	24.1 ^a^	±	14.4	24.9 ^a^	±	9.9	19.3 ^bx^	±	8.9	29.7 ^by^	±	9.4	22.2 ^x^	±	9.0	29.9 ^by^	±	8.9	0.41	<0.0001	0.002
Glucose (g)	23.7 ^a^	±	13.3	25.1 ^a^	±	8.9	19.8 ^bx^	±	8.8	30.2 ^by^	±	7.9	22.2 ^x^	±	8.8	30.9 ^by^	±	7.8	0.18	<0.0001	0.0005
Alcohol (g)	3.2	±	3.6	2.8	±	2.8	2.5	±	2.1	1.9	±	1.7	2.8	±	2.5	2.3	±	1.4	0.07	0.37	0.95
Caffeine (mg)	147.7	±	148.9	103.6	±	114.9	147.9	±	142.7	95.2	±	126.9	150.0	±	149.7	106.6	±	142.7	0.59	0.65	0.73
Bread and cereals (serving)	3.9	±	2.2	4.6	±	2.3	4.0	±	2.0	4.1	±	2.6	4.1	±	2.3	4.1	±	1.9	0.45	0.36	0.26
Fruits (serving)	2.9 ^a^	±	2.6	3.1 ^a^	±	1.6	2.2 ^bx^	±	1.7	6.0 ^by^	±	1.3	2.9 ^x^	±	1.9	5.9 ^by^	±	1.3	<0.0001	<0.0001	<0.0001
Vegetables (serving)	3.6	±	1.9	3.6	±	2.3	3.3	±	1.6	2.6	±	1.6	3.4	±	1.6	3.2	±	2.1	0.02	0.10	0.41
Dairy products (serving)	2.0	±	1.7	2.2	±	1.1	2.0	±	1.3	2.3	±	1.3	1.8	±	0.9	2.2	±	1.4	0.69	0.51	0.74
Animal proteins (serving)	2.3	±	1.2	1.8	±	0.9	2.2	±	1.2	1.8	±	0.8	2.5	±	1.2	2.0	±	1.1	0.13	0.39	0.93
Physical activity (AMI)	298.1	±	147.0	259.0	±	131.9							280.8	±	190.5	218.3	±	159.9	0.13	0.34	0.54

Data are means ± standard deviations. A mixed model adjusted for age, sex, BMI, and baseline values was used to obtain *p*-values. Time effect, treatment effect, and the interaction of treatment by time were significant at ⍺ = 0,05. Significant *p*-values are in bold. Letters stand for significant differences (a,b for time effect and x,y for treatment effect). *N* = 24 for both Rb and control. tx, treatment. AMI, Activity Metabolic Index. tx*Time stands for the interaction between treatment and time effects.

**Table 3:** Differences between follow-up and baseline.

Variable	N	Control			*N*	Rb			*p*-Value
BMI (kg/m^2^)	24	−0.01	±	0.60	24	+0.10	±	0.57	0.43
Waist circumference (cm)	24	−0.18	±	2.18	24	+0.39	±	3.12	0.46
Fasting glucose (mmol/L)	23	+0.01	±	0.35	22	−0.09	±	0.29	0.27
Fasting insulin (pmol/L)	19	+3.10	±	22.98	16	+3.12	±	26.49	0.99
HbA1C (%)	23	−0.03	±	0.13	23	+0.03	±	0.14	0.13
Plasma TG (mmol/L)	22	−0.04	±	0.53	23	−0.17	±	0.68	0.48
ApoB-100 (g/L)	23	+0.01	±	0.09	23	−0.03	±	0.12	0.30
HDL-C (mmol/L)	23	−0.04	±	0.10	23	−0.01	±	0.18	0.43
LDL-C (mmol/L)	22	+0.01	±	0.42	23	+0.03	±	0.42	0.89
CRP (mg/L)	20	+0.90	±	2.33	22	−0.04	±	1.59	0.14
HOMA-IR	13	+0.11	±	0.74	11	−0.16	±	0.53	0.33
Matsuda index	13	−0.60	±	2.08	11	+0.14	±	1.43	0.33

Data are means ± standard deviations. *p*-values were obtained by a general linear model, type III sum of squares. BMI, body mass index. HbA1C, glycated hemoglobin. TG, triglycerides. ApoB-100, Apolipoprotein B. HDL-C, high-density lipoprotein cholesterol. LDL-C, low-density lipoprotein cholesterol. CRP, C-reactive protein. HOMA-IR, homeostatic model assessment of insulin resistance.

**Table 4:** Variations of anthropometrics, body composition, and blood pressure parameters over time within and between groups.

Time	Week 0								Week 4						Week 8						*p*-Values		
Treatment	N Control				N Rb				Control			Rb			Control			Rb			Time	tx	tx*Time
SBP (mmHg)	24	110.8	±	11.2	24	112.8	±	11.0	110.7	±	12.9	113.1	±	10.4	112.8	±	11.9	110.9	±	11.0	0.99	0.37	0.03
DBP (mmHg)	24	68.9	±	8.7	24	71.9	±	8.9	68.8	±	10.4	71.8	±	9.0	69.6	±	11.1	70.3	±	9.3	0.86	0.67	0.34
BMI (kg/m2)	24	29.4	±	3.9	24	30.4	±	4.9	29.5	±	3.9	30.5	±	5.0	29.4	±	3.9	30.5	±	5.1	0.44	0.69	0.69
Waist circumference (cm)	24	98.1	±	11.8	24	98.5	±	13.3	98.5	±	12.1	98.4	±	13.3	97.9	±	12.9	98.9	±	14.1	0.93	0.73	0.23
Hips circumference (cm)	24	108.9	±	7.5	24	112.1	±	10.2	109.5	±	8.4	112.3	±	10.5	109.3	±	8.4	112.1	±	10.5	0.63	0.59	0.77
CRP	20	2.50	±	2.50	21	2.1	±	1.64	2.65	±	2.60	2.86	±	2.59	2.46	±	2.42	3.28	±	2.67	0.30	0.16	0.26
ApoB-100	23	0.86 ^a^	±	0.16	23	0.92	±	0.24	0.95 ^b^	±	0.17	0.92 ^y^	±	0.21	0.87 ^a^	±	0.19	0.89	±	0.25	0.002	0.02	0.03
Total-C (mmol/L)	23	4.53 ^a^	±	0.79	23	4.60	±	0.91	4.85 ^b^	±	0.81	4.70	±	0.84	4.51 ^a^	±	0.88	4.54	±	0.86	0.001	0.14	0.22
HDL-C (mmol/L)	23	1.33	±	0.23	23	1.30	±	0.40	1.39 ^a^	±	0.29	1.32	±	0.35	1.29 ^b^	±	0.27	1.30	±	0.33	0.03	0.49	0.17
LDL-C (mmol/L)	22	2.44 ^a^	±	0.67	23	2.62	±	0.87	2.78 ^b^	±	0.68	2.74	±	0.78	2.45 ^a^	±	0.55	2.65	±	0.82	0.001	0.42	0.14
TG (mmol/L)	22	1.42	±	0.61	23	1.47	±	0.82	1.32	±	0.60	1.39	±	0.64	1.38	±	0.61	1.29	±	0.61	0.37	0.59	0.72
Fasting glucose (mmol/L)	23	4.82	±	0.47	22	5.00	±	0.59	4.85	±	0.49	5.11	±	0.57	4.83	±	0.52	4.91	±	0.57	0.12	0.99	0.25
Fasting insulin (pmol/L)	17	65.3	±	29.0	16	80.1	±	37.4	65.9	±	29.1	77.5	±	33.2	64.0	±	23.2	83.3	±	44.2	0.77	0.59	0.10
HbA1C (%)	23	5.02	±	0.27	23	5.04	±	0.32	4.98	±	0.29	5.03	±	0.30	5.00	±	0.27	5.07	±	0.29	0.11	0.22	0.23

Data are means ± standard deviations. A mixed model adjusted for age, sex, BMI, and baseline values was used to obtain *p*-values (except for BMI and waist circumference, which were adjusted for age, sex, and baseline values only). Time effect, treatment effect, and the interaction of treatment by time were significant at ⍺ = 0.5. Significant *p*-values are in bold. Letters stand for significant differences (a,b for time effect and x,y for treatment effect). tx, treatment. SPB, systolic blood pressure. DBP, diastolic blood pressure. BMI, body mass index. CRP, C-reactive protein. ApoB-100, Apolipoprotein B. Total-C, total cholesterol. HDL-C, high-density lipoprotein cholesterol. LDL-C, low-density lipoprotein cholesterol. TG, triglycerides. HbA1C, glycated hemoglobin. HOMA-IR, homeostatic model assessment insulin resistance. tx*Time stands for the interaction between treatment and time effects.

**Table 5:** List of the most significant differentially expressed genes in Rb group after 8 weeks of Rb supplementation.

RefSeq	Gene Symbol	Gene Name	Nominal *p*-Value	FDR	FC
NM_001114759	*ZNF683*	Zinc finger protein 683	4.5 × 10^−6^	0.07	−1.28
NM_001470	*GABBR1*	Gamma-aminobutyric acid type B receptor subunit 1	7.4 × 10^−6^	0.07	1.22
NM_033423	*GZMH*	Granzyme H	2.5 × 10^−5^	0.12	−1.19
NM_031950	*FGFBP2*	Fibroblast growth factor binding protein 2	2.6 × 10^−5^	0.12	−1.20
NM_030760	*S1PR5*	Sphingosine-1-phosphate receptor 5	4.8 × 10^−5^	0.18	−1.21
NM_139355	*MATK*	Megakaryocyte-associated tyrosine kinase	8.4 × 10^−5^	0.26	−1.16
NM_020395	*INTS12*	Integrator complex subunit 12	9.5 × 10^−5^	0.26	1.39
NM_001144884	*SLC30A7*	Solute carrier family 30 member 7	1.7 × 10^−4^	0.28	1.23
NM_005170	*ASCL2*	Achaete-scute family bHLH transcription factor 2	1.8 × 10^−4^	0.28	−1.16
NM_025069	*ZNF703*	Zinc finger protein 703	1.9 × 10^−4^	0.28	−1.14
NM_001083116	*PRF1*	Perforin 1	2.1 × 10^−4^	0.28	−1.25
NM_138360	*CARMIL3*	Capping protein regulator and myosin 1 linker 3	2.1 × 10^−4^	0.28	−1.29
NM_005601	*NKG7*	Natural killer cell granule protein 7 (1)	2.2 × 10^−4^	0.28	−1.20
NM_001024401	*SBK1*	SH3 domain binding kinase 1	2.2 × 10^−4^	0.28	−1.12
NM_006653	*FRS3*	Fibroblast growth factor receptor substrate 3	2.2 × 10^−4^	0.28	1.17
NR_110601	*PGS1*	Phosphatidylglycerophosphate synthase 1	2.9 × 10^−4^	0.33	1.20
NR_110030	*LINC01215*	Long intergenic non-protein coding RNA 1215	3.0 × 10^−4^	0.33	1.24
NM_001363693	*NKG7*	Natural killer cell granule protein 7 (2)	3.2 × 10^−4^	0.33	−1.24
NM_001145770	*ADGRG1*	Adhesion G protein-coupled receptor G1	3.5 × 10^−4^	0.34	−1.52
NR_024618	*LINC02035*	Long intergenic non-protein coding RNA 2035	3.6 × 10^−4^	0.34	1.14
NM_000234	*LIG1*	DNA ligase 1	3.9 × 10^−4^	0.34	−1.23
NM_001122630	*CDKN1C*	Cyclin dependent kinase inhibitor 1C	3.9 × 10^−4^	0.34	-1.18
NM_170783	*ZNRD1*	Zinc ribbon domain containing 1	4.1 × 10^−4^	0.34	−1.22
NM_013432	*TONSL*	Tonsoku like, DNA repair protein	4.8 × 10^−4^	0.36	−1.15
NM_001145777	*FKBP5*	FKBP prolyl isomerase 5	4.9 × 10^−4^	0.36	1.19
NM_198053	*CD247*	CD247 molecule	5.1 × 10^−4^	0.36	−1.10
NM_032737	*LMNB2*	Lamin B2	5.2 × 10^−4^	0.36	−1.09
NM_004650	*PNPLA4*	Patatin like phospholipase domain containing 4 (1)	6.2 × 10^−4^	0.37	−1.30
NM_001271822	*SERPINB6*	Serpin family B member 6	6.2 × 10^−4^	0.37	−1.17
NM_001358511	*PRDX5*	Peroxiredoxin 5	6.4 × 10^−4^	0.37	−1.29
NM_005686	*SOX13*	SRY-box transcription factor 13	6.4 × 10^−4^	0.37	−1.14
NM_017931	*TTC38*	Tetratricopeptide repeat domain 38	6.4 × 10^−4^	0.37	−1.12
NM_001142389	*PNPLA4*	Patatin like phospholipase domain containing 4 (2)	6.8 × 10^−4^	0.37	1.43
NM_012483	*GNLY*	Granulysin	6.9 × 10^−4^	0.37	−1.18
NM_001004310	*FCRL6*	Fc receptor like 6	6.9 × 10^−4^	0.37	−1.20
NM_024310	*PLEKHF1*	Pleckstrin homology and FYVE domain containing 1	7.2 × 10^−4^	0.37	−1.16
NM_013351	*TBX21*	T-box transcription factor 21	7.3 × 10^−4^	0.37	−1.18
NM_007182	*RASSF1*	Ras association domain family member 1	7.6 × 10^−4^	0.37	−1.14
NM_006056	*NMUR1*	Neuromedin U receptor 1	7.7 × 10^−4^	0.37	−1.13
NM_001110556	*FLNA*	Filamin A	8.9 × 10^−4^	0.42	−1.12
NM_004669	*CLIC3*	Chloride intracellular channel 3	9.3 × 10^−4^	0.42	−1.22
NM_001136044	*TMUB1*	Transmembrane and ubiquitin-like domain containing 1	9.3 × 10^−4^	0.42	−1.11
NM_001335	*CTSW*	Cathepsin W	9.5 × 10^−4^	0.42	−1.14

RefSeq stands for the reference sequence accession number. A negative fold change stands for a decrease in gene expression levels following Rb supplementation. FDR stands for False Discovery Rate-adjusted p-values obtained by paired t-test. FC stands for fold change. Numbers in parentheses in the Gene name column stand for transcript variants.

**Table 6:** List of the plasma metabolites showing significant changes in plasma levels in Rb group after 8 weeks of Rb supplementation.

Metabolite Name	Common Name	Super Pathway	*p*-Value	HMDB
beta-Ala	β-Alanine	Biogenic Amines	0.005	HMDB0000056
TMAO	Trimethylamine N-oxide	Amine Oxides	0.008	HMDB0000925
GDCA	Deoxycholic acid glycine conjugate	Bile Acids	0.01	HMDB00631
CE15:0	Cholesterol 1-pentadecanoate	Cholesterol Esters	0.01	HMDB0060057
TG (22:6/32:0)	1-Palmitoyl-2-palmitoyl-3-docosahexaenoyl-glycerol	Triacylglycerols	0.01	HMDB10418
TG (16:1/32:1)	1-Octadecanoyl-2-(9Z-hexadecenoyl)-3-(9Z-tetradecenoyl)-glycerol	Triacylglycerols	0.02	HMDB0044888
TG (18:2/32:2)	1-Palmitoleoyl-2-palmitoleoyl-3-linoleoyl-glycerol	Triacylglycerols	0.02	HMDB05435
HexCer(d18:1/24:1)	Hexosylceramide	Glucosylceramides	0.02	-
PC aa C42:5	1-Arachidonyl-2-docosapentaenoyl-sn-glycero-3-phosphocholine	Glycerophospholipids	0.04	HMDB0008287
TG (18:0/32:0)	1-Octadecanoyl-2-octadecanoyl-3-(9Z-tetradecenoyl)-glycerol	Triacylglycerols	0.04	HMDB0044753

*p*-values stand for paired *t*-tests. HMDB, Human Metabolome Database.
